# The Investigation of the Diversity of *Lactobacilli* Spp. and Assessment Their Some Probiotic Properties in Traditional Dairy Products in East Azerbaijan Province in Iran

**Published:** 2017

**Authors:** Zeinab Faghfoori, Bahram Pourghassem Gargari, Amir Saber, Maryam Seyyedi, Ahmad Yari Khosroushahi

**Affiliations:** a *Food (Salt) Safety Research Center, School of Nutrition and Food Sciences, Semnan University of Medical Sciences, Semnan, Iran. *; b *Biotechnology Research Center, Tabriz University of Medical Science, Tabriz, Iran.*; c *Department of Biochemistry and Diet Therapy, Faculty of Nutrition, Tabriz University of Medical Sciences, Tabriz, Iran. *; d *Tuberculosis and Lung Research Center, Tabriz University of Medical Sciences, Tabriz, Iran. *; e *Drug Applied Research Center, Faculty of Pharmacy, Tabriz University of Medical Sciences, Tabriz, Iran.*; f *Department of Pharmacognosy, Faculty of Pharmacy, Tabriz University of Medical Sciences, Tabriz, Iran.*

**Keywords:** Probiotics, Lactobacillus, Acid and bile tolerance, Antimicrobial activity, Antibiotic susceptibility

## Abstract

Nowadays, there is an increasing interest in the production of functional foods, particularly probiotic foods. Lactic acid bacteria (LAB) particularly strains of *Lactobacillus* are important bacteria in food microbiology and human nutrition due to their ability to fermented food production and have received considerable attention as probiotics. The traditional fermented dairy foods as a rich source of wild LAB can introduce new *Lactobacillus* strain with probiotic properties into food products. So, the present study was aimed to isolate and identify *Lactobacilli spp*. in traditional dairy products and to assess some of their probiotic properties. For this study, fifty samples including homemade yogurt and cheese were purchased from several rural areas and the intragenic transcribed spacer-PCR (16-23s rDNA) was used for identification of *Lactobacilli*. Some probiotic properties were assayed including resistant to acid and bile, antimicrobial activity, and antibiotic susceptibility.

The isolates were characterized as *L.plantarum*, *L.casei*, *L.paracasei* and *L.rhamnosus*. Out of the fifty-six isolates identified phenotypically as lactobacillus, twenty-four strains were tolerant to pH 2.5 and 0.3% bile salt after 3 h of incubation and only 6 strains showed antimicrobial activity and antibiotic susceptibility. In conclusion, six strains showed potentially probiotic properties including resistant to acid and bile, antimicrobial activity and antibiotic susceptibility. So, we can consider these strains as native probiotic but extra examinations were required for introduction into food products.

## Introduction

Nowadays, the production of functional foods, particularly probiotic foods, is burgeoning due to clarification of positive role of these foods in wellbeing and health (1). Probiotic bacteria are defined as living microorganisms that could confer health benefits on the host when administered in adequate amounts such as the improvement of immune system, modification of intestinal microflora, and anti-pathogenic effects (2, 3). Probiotics are effective in different clinical conditions including infectious diarrhea, necrotizing enterocolitis, antibiotic associated diarrhea, clostridium difficle colitis, helicobacter pylori infections, inflammatory bowel disease, cancer, female uro-genital infection and surgical infections. Therefore, there is growing interest on both basic and clinical sciences in probiotics (4). Some criteria have been identified for a bacterial strain to be a potential probiotic such as the ability to survive and colonization in the gastrointestinal environment, production of anti-bacterial substance, to be of human origin and the possession of at least one beneficial function and assessment of these criteria is an important part for human use (5, 6). Resistance to gastric and bile salt are observed *in-vitro* and are the first host factors that influence strain selection (7, 8).

Lactic acid bacteria (LAB) are a heterogeneous group of gram positive, non-spore-forming bacteria with a rod-shaped or coccoid morphology including *Lactococcus*, *Enterococcus*, *Oenococcus*, *Pediococcus*, *Streptococcus* and *Lactobacillus* that are traditionally and widely used in the production of fermented foods due to their ability to convert sugars into lactic acid (9-11). LAB particularly strains of *Lactobacillus* and *Bifidobacterium* which have received considerable attention as probiotics over the past few years, exist in the human adult gastrointestinal tract and are widely used for a long time especially in dairy and biotechnology industry in production of fermented dairy foods in particular cheese and yogurt and non-dairy products such as vegetables, beverages, and meats. They play key roles in food preservation and contribute to the development of food texture and flavor with health-promoting properties (11). Nowadays, the dairy industry desires to explore new chances for enlarging the diversity of dairy products in order to meet consumers’ demand (12-15). The primary sources of LAB and bifidobacteria are traditional fermented products, breast milk, gastrointestinal tract content, and the feces of human subjects (16). The traditional fermented dairy foods are rich in wild LAB because they are generally produced from raw milk with local microbial flora in a process of spontaneous fermentation without the use of industrial starters. This microflora plays an important role in their specific characteristics and indigenous flavor and texture (11, 17). Isolation and identification of such wild LAB from traditional dairy products can be a good opportunity for the development of new commercial cultures, such as new starter cultures and new probiotics (12).

Over the past few years, there has been increasing commercial interest in *Lactobacillus* supplementation of foods, so it is necessary to introduce new *Lactobacillus* strain with probiotic properties into food products (8). The genus *Lactobacillus*, as a major part of the LAB group, is normally present in the vagina, gastrointestinal tract and is used for the production of yogurt, cheese, sauerkraut, pickles, sourdough, wine, and other fermented products. *Lactobacilli* species are commonly selected as probiotics since they express many important criteria for probiotic selection including high tolerance to acid and bile, ability to adhere to intestinal surfaces, antimicrobial activity, producing exo-polysaccharides and removing cholesterol (18).

Therefore, the aim of the present study was to isolate and identify *Lactobacilli spp*. in traditional dairy products in East Azerbaijan province in Iran and assessment of their some probiotic properties.

## Experimental

*Sampling and isolation of bacteria*

Fifty samples including yogurt and cheese were purchased from several rural areas where dairy products are produced in the traditional way in East Azerbaijan province of Iran from March to September 2014. Samples after collection were immediately placed in an ice box. 5 g of each sample was suspended and homogenized into 10 mL sodium citrate (2% w/v). Afterward, 1 mL of the samples was inoculated with 24 mL of de Man Rogosa and Sharpe (MRS, Merck, Germany) broth and incubated for 48 h at 37 °C. Serial dilutions were prepared from cultures up to 10^-5^, and then 0.02 mL of 10^-5^ dilution was plated onto MRS agar (MRS, Merck, Germany) and incubated under the same conditions in anaerobic condition. After that, single colonies were randomly picked up and incubated in MRS broth. Finally, morphological evaluations were performed of the single colonies. Gram positive, catalase negative and rod-shaped colonies were maintained at -80 °C in MRS containing 20% (v/v) glycerol. We named the isolate as AH and a number, BH and a number or B and a number such as AH-1, AH-2, BH-2, BH-3, BH-7, B-2, B-11, before molecular identification for recognition.

*Identification of the isolates*

Total DNA was extracted from a colony using a DNA extraction kit (Thermo Scientific, USA), according to the manufacturer’s instructions, then checked and visualized via 0.8% agarose gel electrophoresis. The quality and quantity of the extracted DNA were evaluated by the gel monitoring apparatus (Biometra, Gottingen, Germany) and spectrophotometric method respectively. Identification of the isolates was accomplished 16-23Sribosomal DNA (SrDNA) gene sequence. The PCR reaction was performed in a DNA thermal in a thermal cycler PTC 200 (MJC research, Waltham, USA) by using a pair of *Lactobacilli* specific universal primers (Lac F5´CTACACGAAGTCGGAATCGC-3´and Lac R 5´-GTTCCCCCATTCGGAAATCT-3´). The PCR program consisted of the following cycles: 94 ºC for 5 min/Start loop: 35X, 94 ºC for 1 min, 57 or 56 ºC for 1 min, 72 ºC for 1 min, Close loop/ 72 ºC for 10 min. Amplified and purified 16-23SrDNA was sequenced at Bioneer Inc. (Daejeon, Republic of South Korea) and a homology search was performed on the national center for biotechnology information (NCBI) database. Strains with at least 97% homology were considered to belong to the same species.

*Acid and Bile tolerance*

The isolates were grown to close to 10^9 ^cfu/mL in MRS broth at 37 °C and centrifuged for 15 min at 6000×g. The cell pellets were suspended in Phosphate buffer saline (PBS, pH 2.5) and bile (0.3% Oxgall, Sigma), then incubated at 37 °C for 3 h After centrifugation, cells were suspended in 10 mL MRS broth and incubation for 24 h at 37 °C. 1 mL from each solution was serially diluted and a given amount of each dilution (100 µL) was spread-plated onto MRS agar and incubated in the anaerobic condition at 37 °C for 72 h The resistant rate was calculated by comparing treated/untreated cell survival. The survival rate was calculated using the following equation (Table 1):

Survival rate% = (log cfu N1/log cfu N0)× 100

N1= total viable counts of bacterial isolates in the MRS agar after being treated with extra bile salts or in low acidic conditions, N0 = total viable counts of isolates before incubation in harsh conditions (25).

**Table 1:** Acid and bile tolerance of the Lactobacillus isolates after 3 h of incubation at pH 2.5 or 0.3% bile salt.

**Isolate name**	**species**	**Isolation origin**	**pH 2.5** **(log cfu/mL)**					**0.3% bile salt** **(log cfu/mL)**					
			0 h	1 h	2 h	3 h	SR*	0 h	1 h	2 h	3 h	SR*
AH-1	*L. plantarum*	cheese	8.56	8.44	8.31	8.20	95%	8.32	8.14	8.03	7.89	94%
AH-3	*L. plantarum*	cheese	8.13	8.11	8.01	7.93	97%	7.82	7.61	7.31	7.11	90%
AH-4	*L. plantarum*	cheese	7.95	7.84	7.62	7.43	93%	6.75	6.58	6.24	6.17	91%
AH-7	*L. plantarum*	cheese	8.51	8.24	8.10	7.84	92%	7.25	7.05	6.78	6.54	90%
BH-2	*L. plantarum*	cheese	7.68	7.25	7.10	6.92	90%	8.65	8.23	7.87	7.54	87%
BH-7	*L. plantarum*	cheese	8.35	8.12	7.98	7.61	91%	6.38	6.12	5.87	5.67	88%
BH-9	*L. plantarum*	yogurt	8.52	8.24	8.03	7.85	92%	7.72	7.41	7.18	6.98	90%
BH-14	*L. plantarum*	yogurt	8.90	8.71	8.42	8.01	90%	6.57	6.47	6.28	6.16	93%
BH-25	*L. plantarum*	yogurt	7.49	7.12	6.92	6.74	89%	8.82	8.64	8.35	8.19	92%
AH-2	*L. casei*	cheese	6.97	6.78	6.51	6.38	91%	6.73	6.43	6.29	6.05	89%
AH-9	*L. casei*	yogurt	8.87	8.69	8.41	8.23	92%	8.74	8.45	8.15	7.84	89%
B-2	*L. casei*	cheese	7.66	7.51	7.22	7.04	91%	8.35	8.14	7.94	7.67	91%
B-11	*L. casei*	cheese	6.74	6.46	6.32	6.08	90%	7.47	7.24	7.04	6.83	91%
BH-15	*L. casei*	yogurt	6.25	6.15	6.01	5.94	95%	6.43	6.24	6.18	5.79	90%
BH-16	*L. casei*	yogurt	8.88	8.58	8.17	7.91	89%	8.73	8.24	8.01	7.74	88%
BH-32	*L. casei*	cheese	7.54	7.24	7.11	6.97	92%	8.67	8.34	8.21	7.85	90%
BH-35	*L. casei*	yogurt	6.87	6.64	6.43	6.11	88%	6.98	6.54	6.28	5.97	85%
BH-45	*L. casei*	yogurt	8.68	8.27	8.03	7.84	90%	7.84	7.46	7.29	7.14	91%
BH-33	*L.casei*	yogurt	7.68	7.46	7.18	6.97	90%	6.85	6.59	6.35	6.03	88%
B-14	*L. paracasei*	yogurt	6.44	6.14	6.02	5.82	90%	8.69	8.35	8.03	7.78	89%
BH-3	*L. paracasei*	yogurt	7.68	7.35	7.14	6.86	89%	6.21	6.16	5.89	5.67	91%
BH-17	*L. paracasei*	yogurt	8.82	8.52	8.15	7.74	87%	5.85	5.69	5.42	5.02	85%
BH-21	*L. rhamnosus*	cheese	8.92	8.75	8.54	8.31	93%	8.67	8.43	8.27	8.02	92%
BH-53	*L. rhamnosus*	yogurt	7.52	7.23	7.14	6.87	91%	6.74	6.48	6.31	6.10	90%
BH-54	*L. rhamnosus*	yogurt	8.55	8.21	7.94	7.78	90%	7.84	7.54	7.29	6.84	87%

* Survival rate (%) = (log cfu N1/log cfu N0) ×100

**Table 2:** Antimicrobial activity assay of Lactobacillus isolates (mean ± standard error).

**Pathogens**	**Diameter of inhibition zone (mm)**									
	**AH-1**	**AH-9**	**BH-3**	**BH-9**	**BH-21**	**BH-25**	**BH-33**	**BH-45**	**B-11**	**B-14**
*B. cereus*	5.9 ± 0.6	8.4 ± 0.9	9.8 ±0.4	11.3 ± 0.8	7.6 ± 0.7	9.3 ± 0.9	0.0 ± 0.0	8.6 ± 0.5	0.0 ± 0.0	12.4 ± 0.9
*C. albicans*	9.3 ± 0.8	6.4 ± 0.9	0.0 ±0.0	8.0 ± 0.6	11.4 ± 0.5	0.0 ± 0.0	8.6 ± 0.7	13.3 ± 1.2	7.9 ± 0.6	0.0 ± 0.0
*E. coli (026)*	15.5 ± 1.2	12.7 ± 0.9	11.9 ±0.5	14.2 ± 1.2	13.5 ± 0.9	11.9 ± 0.6	10.4 ± 0.7	14.3 ± 1.3	10.6 ± 0.6	12.6 ± 0.8
*E. coli (0157)*	17.0 ± 1.4	13.3 ± 1.3	11.5 ±0.8	14.6 ± 1.1	12.2 ± 0.6	10.3 ± 0.6	11.6 ± 0.7	13.7 ± 0.8	10.6 ± 0.5	11.5 ± 0.7
*E. faecalis*	10.2 ± 0.6	0.0 ± 0.0	8.7 ± 0.6	10.4 ± 0.6	8.5 ± 0.7	0.0 ± 0.0	9.5 ± 0.7	14.4 ± 0.9	12.6 ± 0.7	0.0 ± 0.0
*K. pneumoniae*	12.2 ± 0.5	0.0 ± 0.0	8.6 ± 0.5	10.5 ± 0.5	0.0 ± 0.0	8.6 ± 0.7	0.0 ± 0.0	8.4 ± 0.7	9.2 ± 0.7	10.6 ± 0.6
*L. monocytogenes*	13.0 ± 1.4	9.7 ± 0.7	11.2 ± 0.9	0.0 ± 0.0	10.3 ± 0.5	12.4 ± 0.8	14.4 ± 0.9	0.0 ± 0.0	0.0 ± 0.0	0.0 ± 0.0
*P. aeruginosa*	6.3 ± 0.8	8.5 ± 0.6	0.0 ± 0.0	0.0 ± 0.0	10.3 ± 0.5	0.0 ± 0.0	10.3 ± 0.7	11.8 ± 1.2	0.0 ± 0.0	0.0 ± 0.0
*S. marcescens*	9.5 ± 0.5	11.2 ± 0.8	9.7 ± 0.5	8.4 ± 0.6	0.0 ± 0.0	0.0 ± 0.0	12.3 ± 0.5	0.0 ± 0.0	10.8 ± 0.6	12.5 ± 0.9
*S. saprophyticus*	7.2 ± 0.8	10.3 ± 0.7	0.0 ± 0.0	0.0 ± 0.0	9.6 ± 0.7	8.9 ± 0.6	0.0 ± 0.0	11.5 ± 0.8	11.4 ± 0.9	0.0 ± 0.0
*S. typhimurium*	0.0 ± 0.0	8.0 ± 0.8	7.5 ± 0.5	9.5 ± 0.5	0.0 ± 0.0	0.0 ± 0.0	0.0 ± 0.0	12.3 ± 0.7	8.6 ± 0.6	11.3 ± 0.7
*S. flexneri*	12.3 ± 0.7	14.2 ± 0.6	11.5 ± 1.1	14.4 ± 0.9	16.2 ± 1.2	13.1 ± 0.8	11.3 ± 0.9	15.6 ± 0.7	11.2 ± 0.8	9.8 ± 0.9
*S. mutans*	8.8 ± 0.7	7.9 ± 0.6	10.5 ± 0.8	11.5 ± 0.9	7.5 ± 0.5	0.0 ± 0.0	8.9 ± 0.6	0.0 ± 0.0	0.0 ± 0.0	8.5 ± 0.8
*S. aureus*	0.0 ± 0.0	0.0 ± 0.0	0.0 ± 0.0	0.0 ± 0.0	7.6 ± 0.7	8.4 ± 0.6	0.0 ± 0.0	9.7 ± 0.7	8.7 ± 0.6	13.4 ± 0.9

Strong ≥ 20 mm, Moderate < 20 mm and > 10 mm, and Weak ≤ 10 mm.

**Table 3:** Antibiotic activity assay of *Lactobacillus i*solates by disc diffusion method

**Antibiotic**	**AH-1**	**BH-3**	**BH-21**	**BH-33**	**BH-45**	**B-14**
Ampicillin	S	S	S	S	S	S
Clindamycin	MS	MS	S	S	MS	S
Sulfamethoxazole	S	MS	S	S	S	MS
Chloramphenicol	S	S	MS	S	MS	S
Gentamicin	MS	R	MS	MS	MS	MS
Penicillin	S	S	S	S	S	S
Tetracycline	S	S	MS	S	MS	S
Erythromycin	MS	S	S	MS	S	S
Streptomycin	S	MS	S	MS	S	MS
Vancomycin	R	MS	S	MS	MS	R

S: Sensitive (zone diameter ≥ 17.5); MS: Moderately sensitive (zone diameter 12.5-17.4 mm); R: resistant (zone diameter ≤ 12.4 mm).

**Figure 1:**
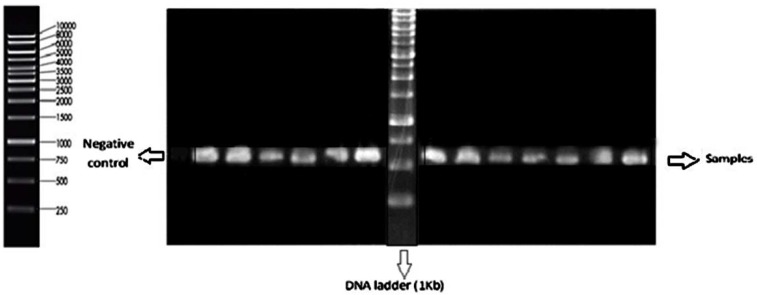
The gel electrophoresis results of polymerase chain reaction (PCR) products *of Lactobacillus* strains on 1% agarose gel

*Antimicrobial activity assay*

The antimicrobial activity of the isolates was assayed against a variety of important human pathogens. Briefly, the isolates were cultured in MRS broth overnight at 37 °C and cell free supernatants were prepared by centrifuging the culture broth at 8000×g for 15 min. The supernatants were adjusted to pH 6.5 and filtered through 0.22 µm membrane filtration, and then 50 µL of each filtrate was added to 7 mm diameter wells, created in the Mueller-Hinton agar plates (Sigma-Aldrich, USA), which before were incubated overnight by indicator pathogens at 37 °C (14). After overnight incubation, the antimicrobial activity was assayed based on the diameter of the clear zones around of the each well (inhibition zone). The antimicrobial activity was divided into three groups: Strong ≥ 20 mm, Moderate < 20 mm and > 10 mm, and Weak ≤ 10 mm (25).

*Antibiotic susceptibility assay*

The antibiotic susceptibility was determined using the disc diffusion method (24). The antibiotic disks including chloramphenicol (30 µg), vancomycin (30 µg), tetracycline (30 µg), erythromycin (15 µg), ampicillin (10 µg), gentamycin (10 µg), clindamycin (2 µg), sulfamethoxazole (25 µg), streptomycin (25 µg) and penicillin (10 µg) were purchased from Padtan Teb Co. (Tehran, Iran).The isolates were cultured on the Mueller-Hinton agar plate then the antibiotic disks were manually placed on plates by using the sterile forceps. After 24 h incubation at 37 °C, the inhibition zone was measured and susceptibility was expressed in terms S: Sensitive (zone diameter ≥ 17.5); MS: Moderately sensitive (zone diameter 12.5-17.4 mm); R: resistant (zone diameter ≤ 12.4 mm) (25).

## Results

Fifty six Gram-positive, catalase-negative, rod-shaped isolates were obtained from traditional dairy products including yogurt and cheese. Twenty four strains were tolerant to pH 2.5 and 0.3% bile salt after 3 h of incubation that sequenced On the basis of the 16-23SrDNA gene analysis (Figure 1), thus they were selected for antibacterial and antibiotic susceptibility examinations. The isolates were characterized as *L.plantarum* (36 %), *L.casei* (40%), *L.paracasei* (12%), and *L.rhamnosus* (12%) (Table 1).

*Antimicrobial activity*

Table 2 presents the antimicrobial activity. The antimicrobial activity assay was conducted against the indicator strains, including *Bacillus cereus* subsp. *Kenyae* (Persian type culture collection, Tehran, Iran. [PTCC 1539]), *Candida albicans* (PTCC 5027), native isolate of *E.coli* (026), *E.coli 0157* (PTCC 1276), *Enterococcus faecalis* (PTCC 1394), *Klebsiella pneumonia* (PTCC 1053), *Listeria monocytogenes* (PTCC 1163), *Pseudomonas aeruginosa* (PTCC 11811), *Serratia marcescens* (PTCC 1187), *Staphylococcus saprophyticus* (PTCC 1440), *Salmonella typhimurium* (American type culture collection, Virginia, USA [ATCC 14028]), *Shigella flexneri* (PTCC 1234), *Streptococcus mutans* (PTCC 1683) and *Staphylococcus aureus* (ATCC 25923). Ten *Lactobacillus* isolates showed weak to moderate inhibitory activities against the indicator bacteria, although the extents of inhibitory effects were variable. The highest inhibitory activity was found against the *E.coli* pathogens and all ten isolates had moderate inhibitory effects against them.

*Antibiotic susceptibility*

The antibiotic susceptibility of ten isolates with antimicrobial activity against the antibiotics was evaluated using measurement of the inhibition zone diameter. Table 3 lists the antibiotic susceptibility results of the *Lactobacillus* isolates. Based on our findings, only six isolates including AH-1 (*L.plantarum*), BH-3 and B-14 (*L.paracasei*), BH-21 (*L.rhamnosus*) and BH-33 and BH-45 (*L.casei*) were sensitive or semi-sensitive to the majority of antibiotics. All six strains were sensitive toward penicillin and ampicillin. AH-1 and B-14 were resistant to vancomycin, and BH-3 was resistant to gentamicin.

## Discussion

Probiotic bacteria are recently used in different health-related areas such as the control of inflammation and infections, management of allergic diseases, antibiotic-related diarrhea, gastroenteritis, constipation, lactose intolerance, a preventive role in the onset of tumors (19). *Lactobacilli* are widely spread in nature and are among the bacteria, most commonly used as probiotics in the food industry (20, 21). Traditional fermented dairy foods such as various cheeses or yogurts are good reserves for finding new probiotics in particular the genus of *Lactobacilli* (20). Up to now, many efforts have been made to identify potential probiotics from several sources such as humans or fermented foods. It has been considered that only a strain of human origin could ensure a probiotic effect in humans, since bacteria are often isolated from the intestinal flora may have originated from ingested foods, therefore the isolation microflora from fermented milk products may be a good approach for overcoming to this problem (21).

To act as a probiotic and to exert beneficial effect, lactic acid bacteria must be able to survive the acidic conditions in the stomach and resist bile acids at intestine. The pH of gastric juice is usually 3.0, but fluctuates with diet considerably (1.5-4.5) and bile salts concentration is ranging from 0.03% to 0.3%. These characteristics play a fundamental role in the vitality of probiotic bacteria (8). In the present study, we assayed the acid tolerance of isolated bacteria in pH 2.5 and bile tolerance in 0.3% bile salt. Out of the fifty-six isolates identified phenotypically as *Lactobacillus*, thirty-two isolates indicated reduced viability after being exposed to pH 2.5 and 0.3% bile salt. In Wang *et al.* study (14) most of the isolates were sensitive to pH 2.

Due to the increasing use of *Lactobacillus* species as probiotics, the accurate classification of these bacteria looks to be necessary. The identification based on phenotypic methods is difficult and time consuming. Molecular methods such as species-specific PCR, 16-23SrDNA sequencing, pulsed-field gel electrophoresis (PFGE) of rare-cutting restriction enzyme fragments, amplified ribosomal DNA restriction analysis (ARDRA) and DNA-DNA hybridization, have proven to be more reliable and improve the identification of *Lactobacilli* (22). The intragenic transcribed spacer-PCR (ITS-PCR) offered in this study for identification of *Lactobacilli*. It is based on amplification of sequences located between conserved genes encoding the 16-23SrDNA. The evidence offers that the 16-23SrDNA targeted PCR assay can be appropriate as reliable identification tool for the closely related *Lactobacilli* because these sequences show a high degree of sequence and length variation at the genus and species levels, so could be used not only for interspecies, but also for intraspecies, identification of *Lactobacillus* strains (22, 23).Antibiotics are an important tool for fighting with bacterial infections, therefore antibiotic resistance can be a significant hazard for many people. Recently many investigators have exhibited that commensal bacterium including LAB may have some antibiotic resistance genes similar to those found in human pathogens which can transfer these genes to pathogenic bacteria. Genes conferring resistance to tetracycline, erythromycin and vancomycin have been detected in some species isolated from fermented meat and milk products (24). In this study, we observed resistant to vancomycin in some isolates and high susceptibility to penicillin and ampicillin. The isolates that assessed in this study, showed also a high susceptibility toward erythromycin and tetracycline. Gad and coworkers study revealed high susceptibility of LAB isolates to ampicillin and amoxicillin and high resistance rate to vancomycin (15). In conclusion, probiotic effects are strain and species specific and precise screening is required for selection of truly probiotic *Lactobacilli* (20, 21). Of the 24 isolated strains that were traditionally characterized to be *Lactobacillus* according to phenotypic traits and molecular identification, 6 strains showed potentially probiotic properties including resistant to acid and bile, antimicrobial activity and antibiotic susceptibility.
